# Phase Angle as a Non-Invasive Biomarker of Fluid Overload in Canine Right Heart Failure: A Bioelectrical Impedance Approach

**DOI:** 10.3390/ani15192877

**Published:** 2025-09-30

**Authors:** Zongru Li, Ahmed S. Mandour, Ahmed Farag, Tingfeng Xu, Kazuyuki Terai, Kazumi Shimada, Lina Hamabe, Aimi Yokoi, Ryou Tanaka

**Affiliations:** 1 Veterinary Teaching Hospital, Tokyo University of Agriculture and Technology, Tokyo 183-8509, Japan; 2 Department of Animal Medicine (Internal Medicine), Faculty of Veterinary Medicine, Suez Canal University, Ismailia 41522, Egypt; dr_mandour@vet.suez.edu.eg

**Keywords:** phase angle, right heart failure, extracellular fluid, bioelectrical impedance analysis, InBody M20

## Abstract

Background: Right heart failure (RHF) in dogs is marked by pathological fluid redistribution and extracellular fluid (ECF) accumulation, which intensifies cardiac work-load and disrupts systemic homeostasis. This study aimed to validate the clinical utility of phase angle (PhA), a key biomarker derived from bioelectrical impedance analysis (BIA), as a non-invasive and real-time indicator of fluid distribution abnormalities in canine RHF. PhA reflects cellular integrity and fluid balance, making it a promising tool for detecting ECF accumulation, one of the hallmark features of RHF. Additionally, the study assessed the feasibility and clinical applicability of the InBody M20 device in veterinary cardiology, supporting its potential role in monitoring and managing fluid-related complications in dogs with RHF. Methods: A total of 110 canine patients presenting to the Tokyo University of Agriculture and Technology Veterinary Hospital were enrolled and categorized into three groups: right-sided heart failure (RHF), left-sided heart failure (LHF), and healthy controls. Phase angle (PhA) was measured using the InBody M20 device, and plasma osmolality (OSM) was also assessed. Additionally, the effects of body weight and age on PhA values were analyzed to account for potential confounding factors. Results: Dogs in the RHF group exhibited significantly lower phase angle (PhA) values and higher plasma osmolality (OSM) compared to those in the LHF and control groups. A strong positive correlation was observed between PhA and OSM (r = 0.9211, *p* < 0.0001). Additionally, PhA measured at 5 kHz demonstrated a significant negative correlation with body weight (r = –0.4536, *p* = 0.0007), while PhA at 50 kHz showed a significant negative correlation with age (r = –0.3219, *p* = 0.0176). Conclusions: PhA is a reliable and non-invasive biomarker for assessing extracellular fluid accumulation and diagnosing right heart failure in dogs. Its strong correlation with plasma osmolality, as well as its associations with body weight and age, highlights its clinical relevance for comprehensive fluid status evaluation. The findings support the feasibility and applicability of using the InBody M20 device in veterinary cardiology to monitor and manage fluid-related complications in canine patients.

## Simple Summary

Heart failure is a common and serious condition in dogs, but monitoring changes in fluid levels is often difficult without invasive tools. This study investigated a noninvasive device that sends a weak electrical current through the dog’s body to measure a value called “phase angle”, which may reflect the amount of fluid inside and outside cells. We focused on dogs with right heart failure, a condition that causes fluid accumulation. Comparing dogs with heart failure with healthy dogs, we found that the phase angle was lower in the affected group and significantly correlated with blood test results related to fluid balance. These findings suggest that this approach could help veterinarians monitor dogs with heart failure more safely and easily. In the future, this approach may reduce the need for more invasive testing and enable faster adjustments to treatment plans. This represents a step toward improving quality of life and outcomes for dogs with heart disease.

## 1. Introduction

Congestive heart failure is characterized by venous congestion and extracellular fluid accumulation in various organs and body cavities, depending on the affected side. In particular, evaluating fluid distribution and its clinical implications has become a critical focus in both human and veterinary cardiology. Bioelectrical impedance analysis (BIA) offers a rapid, non-invasive, and cost-effective method to assess body composition and fluid compartments, including intra- and extracellular water distribution, using resistance and reactance measurements [1]. Recent human studies have emphasized the prognostic value of phase angle (PhA), a parameter derived from BIA, highlighting its associations with muscle strength, quality of life, and survival across various clinical settings [2].

In human medicine, bioelectrical impedance analysis (BIA) has become a valuable tool for monitoring fluid retention, a critical factor in the management of heart failure [2]. BIA offers a non-invasive, cost-effective method for assessing body composition and fluid shifts, and its applications have expanded to evaluating clinical status across a range of conditions, including cardiac diseases [3]. Key parameters measured by BIA, such as total body water (TBW) and extracellular water (ECW), have been shown to correlate with the severity of heart failure, reinforcing its prognostic value [4,5]. These insights underline the growing clinical relevance of BIA in guiding the management and monitoring of patients with heart failure.

PhA serves as a reliable indicator of cellular integrity and fluid distribution. Studies show that interventions enhancing cellular health, such as resistance training, can significantly increase PhA, reflecting improved cell function and hydration [6]. Clinically, reduced PhA values have been linked to higher rates of frailty and increased long-term mortality, independent of comorbidities [7]. Additionally, PhA is influenced by factors such as age, sex, and body composition, which must be considered in clinical evaluation [8]. Given its sensitivity to physiological changes and its strong prognostic value, PhA represents a promising tool for assessing disease severity and monitoring outcomes in chronic conditions like heart failure. Despite these advances, the clinical utility of BIA and PhA for monitoring fluid imbalance in canine heart failure, particularly right-sided heart failure, has not been fully explored.

In healthy young males, a six-month program of regular physical training resulted in significant improvements in PhA, accompanied by increases in lean soft tissue and bone mineral content and reductions in blood glucose levels, suggesting that PhA reflects dynamic changes in cellular health and metabolic regulation beyond simple body composition measures [9]. Similarly, in elderly individuals, PhA was independently associated with higher Global Cardiovascular Risk Scores, indicating its role as a prognostic marker for cardiovascular health independent of age and traditional anthropometric factors [10]. Although most research on phase angle has been conducted in human populations, the findings provide valuable insights potentially translatable to veterinary cardiology. One study validated BIA’s ability to detect changes in body fluid volumes in dogs, particularly after blood donation, showing significant changes in bioelectrical parameters in donor dogs compared to controls [11]. This suggests that BIA, including phase angle (PhA), could be a valuable tool for monitoring fluid balance in veterinary practice. Similarly, multi-frequency bioelectrical impedance analysis (MF-BIA) has been explored for its ability to assess body composition in cats, including total body water, extracellular water, and intracellular water. By comparing MF-BIA results with established methods like deuterium and bromide space, this study highlights its potential to enhance clinical monitoring and body fluid management in feline patients [12]. These findings highlight the potential of PhA as a non-invasive, sensitive indicator of cellular integrity, hydration status, and cardiovascular risk, offering promising applications for monitoring disease progression and therapeutic response in veterinary cardiac patients.

This study aims to investigate the potential of the non-invasive InBody M20 device for assessing fluid distribution in dogs with right-side heart failure. By examining the relationship between phase angle (PhA) and plasma osmolality (OSM), we hypothesize that PhA can serve as a valuable indicator of fluid balance in animals with various heart conditions. This cross-sectional study will compare PhA and OSM values in dogs diagnosed with left-side heart failure, right-side heart failure, and healthy controls. with the objective of assessing the feasibility and diagnostic utility of the InBody M20 device in routine veterinary practice. Through this investigation, we seek to establish a foundation for incorporating this technology into the management of heart failure in animals while contributing to the development of non-invasive diagnostic tools for veterinary care.

## 2. Materials and Methods

### 2.1. Inclusion and Exclusion Criteria

This study retrospectively included client-owned dogs who presented to our veterinary hospital for their first consultation between December 2023 and August 2025. All cases were non-consecutive and non-overlapping. Dogs were considered for inclusion if they showed clinical signs suggestive of congestive heart failure (CHF), such as exercise intolerance, coughing, tachypnea, or dyspnea at rest.

To ensure diagnostic consistency, each case was initially evaluated by the attending clinician and subsequently discussed by two board-certified cardiology specialists. The final diagnosis was based on a combination of clinical signs, echocardiographic findings, and thoracic radiographs. Dogs were then classified into right-sided heart failure (RHF), left-sided heart failure (LHF), or control groups based on this comprehensive evaluation.

RHF was diagnosed in dogs with clinical signs of systemic venous congestion (e.g., ascites, jugular vein distention) confirmed by echocardiographic evidence of right ventricular enlargement, tricuspid regurgitation, or pulmonary stenosis.

LHF was diagnosed in dogs with signs of pulmonary edema (e.g., coughing, dyspnea) supported by echocardiographic findings such as left atrial/ventricular enlargement or mitral regurgitation.

Dogs with ambiguous diagnoses (e.g., suspected biventricular failure), incomplete records, or subclinical disease were excluded. Only dogs meeting the moderate or severe criteria listed in Table 1 were included for analysis; mild cases were excluded to reduce variability.

Control dogs were selected from first-visit cases diagnosed with non-systemic, non-cardiac conditions such as orthopedic or ophthalmologic issues. All control animals underwent complete physical examination, echocardiography, and routine blood biochemistry to rule out underlying cardiac or systemic diseases, including anemia, renal dysfunction, or endocrine disorders.

Dogs receiving active diuretics or intravenous fluid therapy at the time of BIA measurement were excluded to avoid confounding effects. Additional exclusion criteria included clinical dehydration, cachexia, neoplasia, or poor tolerance of lateral recumbency due to respiratory distress.

All BIA measurements were performed under standardized conditions following an 8 h fasting period.

### 2.2. Heart Failure Definition

In this study, heart failure was defined based on a combination of diagnostic imaging and clinical signs of fluid overload.

Right-sided heart failure (RHF) was diagnosed when right-sided cardiac diseases (e.g., tricuspid regurgitation, pulmonary stenosis, pulmonary regurgitation) were accompanied by clinical evidence of systemic venous congestion, such as ascites, hepatomegaly, or jugular vein distension.

Left-sided heart failure (LHF) was diagnosed when left-sided cardiac diseases (e.g., mitral regurgitation, aortic stenosis, patent ductus arteriosus) were accompanied by signs of pulmonary congestion, including pulmonary edema on thoracic radiographs or clinical symptoms such as coughing, dyspnea, or tachypnea.

Dogs with mild structural cardiac abnormalities but without any signs of fluid retention were not classified as having heart failure and were excluded from the RHF or LHF groups.

### 2.3. Cardiac Disease Classification Criteria

RHF was diagnosed based on evidence of right atrial and/or right ventricular dysfunction with systemic venous congestion. Common abnormal findings included tricuspid regurgitation (TR), pulmonary artery stenosis (PS), or pulmonary artery regurgitation (PR), confirmed through sight side enlargement and elevated right-sided pressures. Clinical signs such as ascites and peripheral edema supported RHF diagnosis.

LHF was defined by left atrial enlargement (LA/Ao > 1.5), pulmonary venous congestion, and associated conditions including mitral regurgitation (MR), patent ductus arteriosus (PDA), or aortic stenosis (AS). Typical clinical presentations involved exercise intolerance, respiratory distress, or pulmonary edema.

### 2.4. Bioelectrical Impedance Monitoring

Bioelectrical impedance analysis (BIA) was performed using the InBody M20 device (InBody Co., Ltd., Seoul, Republic of Korea), a compact, portable multi-frequency analyzer. The device includes a built-in battery, allowing up to 4 h of operation without external power supply, making it suitable for clinical use in various veterinary environments. Phase angle (PhA) was derived from raw impedance values at fixed frequencies of 5, 50, and 250 kHz. Due to differences in cellular penetration at each frequency, PhA values reflect different physiological characteristics.

**Figure 1:**
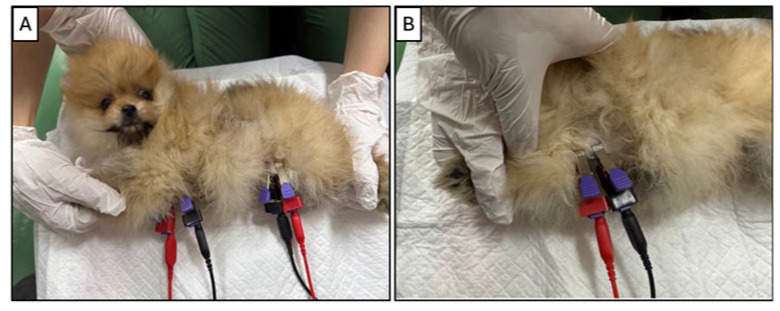
(**A**) Veterinary ECG clip electrodes were used for all measurements. The black electrodes were placed at the left axilla (front limb) and left iliac region (hind limb) to capture impedance data across the trunk region. (**B**) Proper electrode placement required direct skin contact. Hair removal was performed when necessary to ensure optimal conductivity and signal quality.

Measurement Protocol: All measurements were conducted under standardized environmental conditions, including temperature control and reduced ambient electrical interference. Dogs were positioned in right lateral recumbency on a non-conductive examination table. Veterinary ECG clip electrodes were used for all measurements. Operators wore insulated gloves to minimize background noise and ensure reproducibility (Figure 1). Each measurement was repeated twice, and the average value was used for analysis.

To ensure the reliability and repeatability of the measurements, each dog underwent bioelectrical impedance analysis (BIA) at least three times during the same clinical visit. For each measurement, the InBody M20 device performed 50 impedance readings over a 2.5 s interval (sampling at 50 ms). The average of these 50 readings was automatically calculated and recorded as the representative phase angle (PhA) value. Among the repeated trials, the most stable set of data was selected for further analysis. This protocol was designed to minimize the influence of transient artifacts and measurement variability while maintaining clinical feasibility in a veterinary setting.

### 2.5. Plasma Osmolality Assessment

Venous blood samples were collected in a fasted state on the same day as BIA measurements. Plasma osmolality (OSM) was calculated using the following formula:

OSM (mOsm/kg) = 2 [Na^+^] + ([Glucose]/18) + ([BUN]/2.8) (1)

All samples were analyzed using an automated clinical chemistry analyzer.

### 2.6. Echocardiographic Assessment

**Figure 2:**
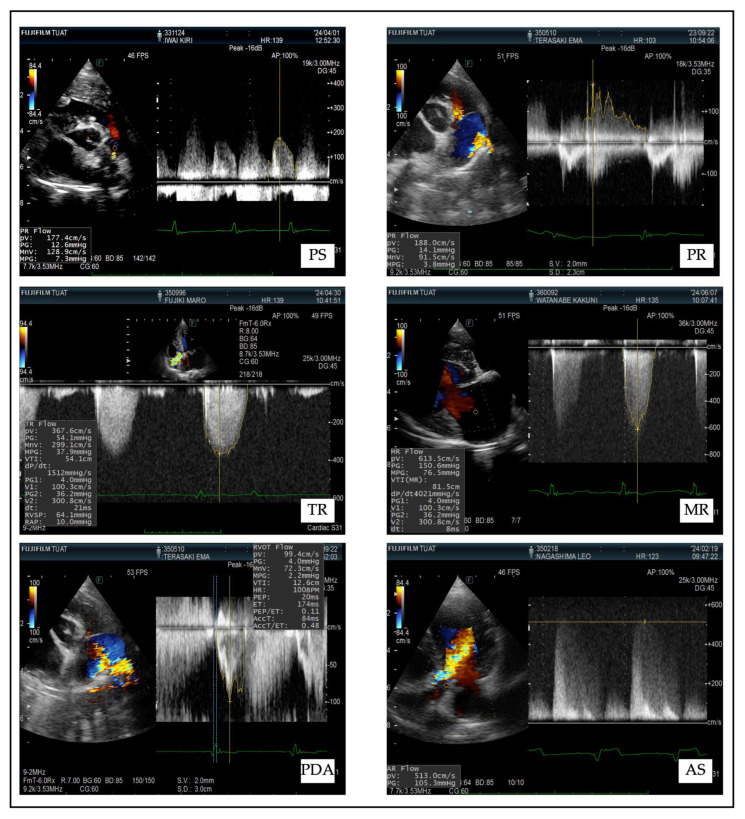
Pulmonary stenosis (PS): Narrowing at the level of the pulmonary valve or subvalvular region is evident, with aliasing and increased flow velocity on Doppler imaging. Pulmonary regurgitation (PR): Diastolic retrograde flow through the pulmonary valve is visualized on color Doppler, confirming valve incompetence. Tricuspid regurgitation (TR): Doppler imaging reveals systolic backflow from the right ventricle to the right atrium, consistent with tricuspid insufficiency. Patent ductus arteriosus (PDA): Color Doppler imaging reveals continuous turbulent flow from the aorta to the pulmonary artery, consistent with a left-to-right shunt. Aortic stenosis (AS). Color Doppler demonstrates turbulent systolic flow across a narrowed aortic valve, and spectral Doppler confirms elevated pressure gradient. Mitral regurgitation (MR): Regurgitant jet from the left ventricle to the left atrium is visible during systole, indicating mitral valve insufficiency.

Comprehensive echocardiographic and Doppler ultrasonographic evaluations were performed using a standardized imaging protocol. Left atrial size (LA/Ao), ventricular function, and valvular abnormalities were assessed to confirm the diagnosis and assign the cardiac condition category. Severity of regurgitant or stenotic lesions was quantified via pressure gradients and Doppler velocities (Table 1). Representative echocardiographic images corresponding to each disease group (PDA, PR, PS, AS, MR, and TR) are provided in the supplementary figures to illustrate the diagnostic features and grouping criteria applied in this study (Figure 2).

### 2.7. Statistical Analysis

Statistical analyses were performed using GraphPad Prism (Version 10.2.1, GraphPad Software Inc., San Diego, CA, USA). Normality of distributions was assessed via Q–Q plots, and Levene’s test confirmed homogeneity of variances. One-way ANOVA followed by Tukey’s multiple comparison test was used to evaluate differences in PhA and OSM between the three groups.

Pearson correlation analysis was used to assess relationships between PhA, OSM, age, and body weight. Statistical significance was defined as *p* < 0.05. Sample size was not predetermined; eligible cases were consecutively included until trends in data saturation were observed.

## 3. Results

### 3.1. Study Population

A total of 110 client-owned canine patients were prospectively enrolled from the Veterinary Medical Teaching Hospital at Tokyo University of Agriculture and Technology, Tokyo, Japan. All dogs underwent comprehensive clinical examinations and were stratified into three groups based on clinical signs and echocardiographic findings: right-sided heart failure (RHF; *n* = 35), left-sided heart failure (LHF; *n* = 35), and healthy controls (*n* = 40).

Inclusion criteria consisted of adult dogs with confirmed diagnoses of heart failure or those deemed clinically healthy by routine cardiac screening. Exclusion criteria included systemic illnesses (e.g., renal failure, neoplasia), dermatological lesions interfering with electrode placement, and severe musculoskeletal conditions that precluded safe recumbency during measurements. Heart failure was defined and classified using standard echocardiographic and Doppler ultrasonography criteria (Table 1).

### 3.2. Patient Characteristics and Data Availability

A total of 110 canine patients were enrolled and categorized into three groups: right-sided heart failure (RHF, *n* = 35), left-sided heart failure (LHF, *n* = 35), and healthy controls (*n* = 40), based on echocardiographic and clinical diagnostic criteria. However, the number of cases included in each specific analysis varied due to the following reasons:

1. A subset of RHF and LHF cases demonstrated overlapping biventricular dysfunction and were excluded from strictly categorized comparisons.
2. Several dogs exhibited systemic comorbidities not directly related to cardiac dysfunction, which could have confounded bioimpedance and biochemical results.
3. Incomplete datasets were present in some individuals while PhA measurements were available, corresponding plasma biochemical parameters required to calculate osmolality (OSM) were missing.

These factors led to slight variations in sample sizes across different analyses (Table 2). Nonetheless, consistent trends and statistically significant findings emerged, supporting the robustness of the results.

### 3.3. Differences in Plasma Osmolality and Phase Angle Between Groups

**Figure 3:**
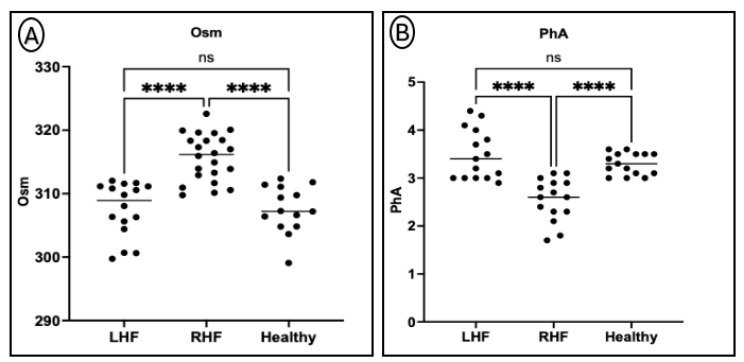
(**A**) Plasma osmolality (OSM) was significantly higher and phase angle (PhA) significantly lower in the RHF group compared to LHF and healthy controls. (**B**) No significant differences were observed between the LHF and control groups for either parameter. ****: *p* < 0.0001; ns: not significant.

Plasma OSM levels were significantly elevated in the RHF group compared to both the LHF and healthy control groups (*p* < 0.0001), indicating pronounced extracellular fluid accumulation. In contrast, PhA values were significantly reduced in the RHF group relative to the LHF and control groups (*p* < 0.05), suggesting impaired cellular membrane integrity and altered fluid balance. No significant differences were detected between the LHF and control groups for either parameter, indicating that left-sided failure may exert less systemic impact on fluid distribution compared to right-sided failure (Figure 3).

### 3.4. Correlation Between Plasma Osmolality and Phase Angle

**Figure 4:**
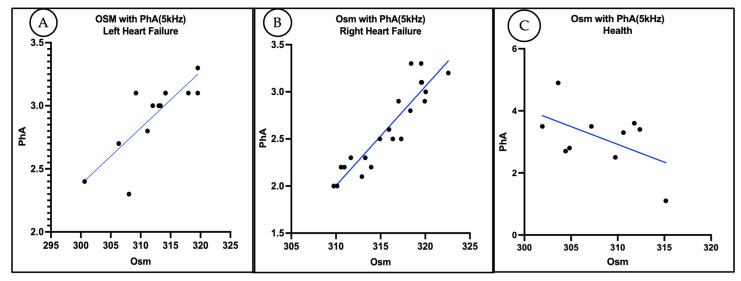
(**A**) Scatter plot showing a significant positive correlation between plasma osmolality (OSM) and phase angle (PhA) in dogs with left-sided heart failure (LHF). (**B**) Scatter plot illustrating a stronger correlation between OSM and PhA in dogs with right-sided heart failure (RHF). (**C**) The scatter plot shows that there is no correlation between OSM and PhA in healthy (Health) dogs.

Strong positive correlations were observed between PhA and OSM within both heart failure subgroups (RHF group: r = 0.9211, *p* < 0.0001, R^2^ = 0.8484; LHF group: r = 0.8138, *p* = 0.0004, R^2^ = 0.6622) (Figure 4), supporting the physiological relationship between systemic fluid distribution and cellular health. These findings highlight OSM as a robust predictor of PhA, with the RHF group demonstrating a notably stronger association. In contrast, the healthy control group showed a moderate but statistically non-significant negative correlation between PhA and OSM (r = −0.5175, *p* = 0.1255, R^2^ = 0.2679; Figure 4). Although a trend was observed, the lack of significance suggests that the relationship between systemic fluid balance and cellular integrity may not be as pronounced in healthy individuals compared to those with heart failure.

### 3.5. Relationship Between Body Weight and Phase Angle

**Figure 5:**
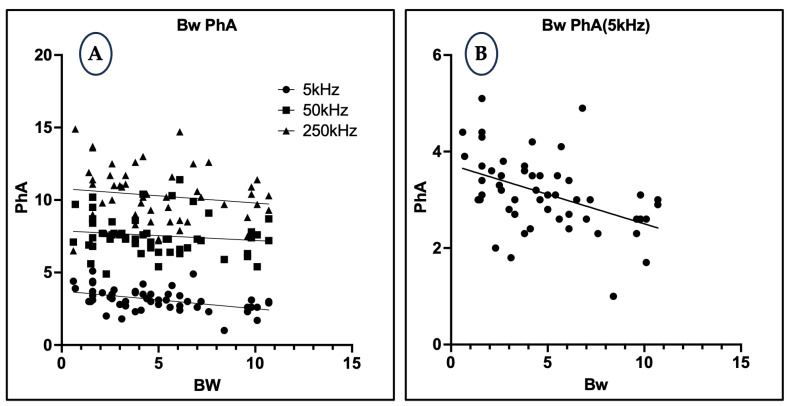
(**A**) Scatter plot showing the relationship between body weight (BW) and PhA at 5 kHz, 50 kHz, and 250 kHz. (**B**) Linear regression plot highlighting the significant negative correlation at 5 kHz only.

At a low frequency of 5 kHz, PhA demonstrated a significant negative correlation with body weight (r = −0.4536, *p* = 0.0007, R^2^ = 0.2057), suggesting that heavier animals had a relatively higher proportion of extracellular water and consequently lower PhA values. However, at higher frequencies—50 kHz (*p* = 0.3192, R^2^ = 0.01984) and 250 kHz (*p* = 0.2922, R^2^ = 0.02216)—which are more reflective of intracellular fluid compartments, correlations were weak and not statistically significant, indicating a limited association between body weight and intracellular fluid distribution (Figure 5).

### 3.6. Relationship Between Age and Phase Angle

**Figure 6:**
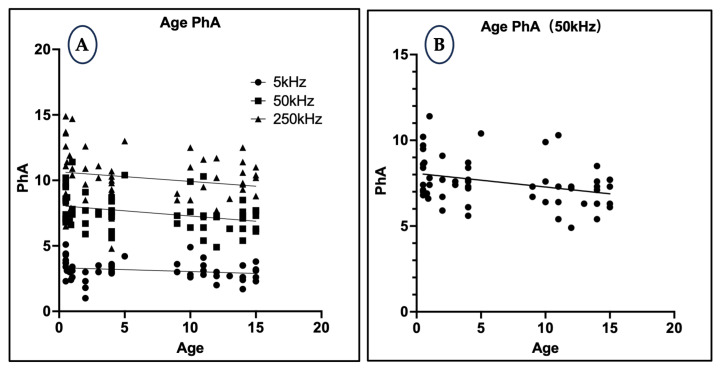
(**A**) Scatter plot illustrating age vs. PhA at 5 kHz, 50 kHz, and 250 kHz. (**B**) Focused plot at 50 kHz, showing a significant negative correlation with age.

At 50 kHz, PhA showed a statistically significant negative correlation with age (r = −0.3219, *p* = 0.0176, R^2^ = 0.1036), reflecting age-related declines in cell mass, membrane function, and intracellular fluid volume. In contrast, correlations at 5 kHz and 250 kHz were not significant (R^2^ < 0.05, *p* > 0.1), indicating that the 50 kHz measurement may be more sensitive for detecting physiological aging changes (Figure 6).

## 4. Discussion

This study investigated the potential of phase angle (PhA), a raw parameter derived from bioelectrical impedance analysis (BIA), as a noninvasive indicator of heart failure severity in canine patients, particularly in cases of right heart failure (RHF). PhA is widely recognized as a marker of cellular integrity and hydration status, reflecting the distribution of intracellular and extracellular fluids [2,3,23].

In the current study, we seek to establish a foundation for incorporating BIA technology into the management of heart failure in animals while contributing to the development of non-invasive diagnostic tools for veterinary care.

In our findings, dogs with RHF exhibited significantly lower PhA values and elevated OSM compared to both LHF and healthy control groups, consistent with systemic extracellular fluid accumulation and compromised cell membrane function. Moreover, variations in PhA are known to be influenced by factors such as age, BMI, and disease state, underscoring the need for context-specific reference values and interpretation [23]. While PhA alone cannot definitively distinguish fluid overload from other pathophysiological processes, the integration of biochemical markers such as plasma OSM provides a complementary validation, enhancing diagnostic specificity [3]. This dual-parameter approach may offer a valuable tool for evaluating systemic fluid balance and cellular health in veterinary cardiology.

Right heart failure (RHF) frequently arises secondary to pulmonary hypertension, pulmonic or tricuspid valvular disorders, or intrinsic myocardial dysfunction, leading to impaired right ventricular output and systemic venous congestion [24]. This hemodynamic compromise triggers a cascade of compensatory mechanisms, including neurohormonal activation and fluid retention, ultimately resulting in extracellular fluid (ECF) expansion and clinical signs such as peripheral edema and ascites, particularly in advanced stages [24,25]. Experimental models have shown that in low-output heart failure, such as that induced by chronic tachypacing, a marked increase in pulmonary arterial and capillary pressures coincides with fluid retention and hormonal dysregulation, including inappropriate secretion of antidiuretic hormone (ADH) despite elevated plasma osmolality [26], thereby contributing to dilutional hypo-osmolality and exacerbated congestion [25].

This pathophysiological pattern parallels findings in our RHF group, where elevated plasma osmolality and reduced phase angle (PhA) likely reflect both cellular dysfunction and fluid overload. Furthermore, the association between PhA and cardiovascular risk observed in elderly human populations reinforces the potential value of PhA as a surrogate marker of systemic decompensation and overall cardiovascular burden in RHF [10].

In our study, plasma osmolality (OSM) was elevated in dogs with right heart failure (RHF), which appears inconsistent with the expected reduction during acute water retention. One possible explanation is that most RHF cases included in this study were in a compensated or chronic stage rather than experiencing acute decompensation. In such conditions, sustained neurohormonal activation, including the renin–angiotensin–aldosterone system (RAAS) and antidiuretic hormone (ADH), may contribute to altered sodium and water handling. A recent study reported that dogs with stage D congestive heart failure exhibited a notable discrepancy between measured and corrected serum chloride levels, suggesting the presence of relative water excess [27]. Although OSM was not directly evaluated in that study, the authors proposed a possible link with ADH activity. These findings, together with the reported RAAS activity pattern in dogs with refractory CHF [28], may help explain the elevated OSM values observed in our RHF group and underscore the complexity of fluid regulation in chronic heart failure.

Left heart failure (LHF) primarily disrupts pulmonary venous flow due to impaired left ventricular function, resulting in pulmonary congestion and edema rather than generalized extracellular fluid (ECF) accumulation [29,30,31]. This pulmonary-specific fluid overload is largely confined to the lung interstation and alveoli, often manifesting clinically as dyspnea, orthopnea, and pulmonary rales, rather than peripheral edema. The pathophysiology of LHF involves elevated left atrial and pulmonary capillary pressures, stimulating neurohormonal systems—such as the renin–angiotensin–aldosterone system (RAAS) and antidiuretic hormone (ADH)—that can promote fluid retention [24,25,31]. However, in LHF, this retention tends to be redistributed to pulmonary circulation rather than contributing to total body water excess. As a result, systemic biomarkers such as plasma osmolality (OSM) and phase angle (PhA)—which reflect overall hydration and cellular integrity—may remain unchanged in LHF compared to healthy controls [2,3,25,32]. These findings underscore the compartmentalized nature of fluid overload in LHF and highlight that PhA and OSM may be more sensitive markers of systemic rather than pulmonary fluid shifts.

The strong positive correlation between PhA and OSM in RHF (r = 0.9211, *p* < 0.0001) further supports the utility of PhA as a sensitive and non-invasive indicator of systemic fluid status [6,30,33]. This relationship likely reflects the hemodynamic alterations and fluid accumulation in the extracellular compartment commonly observed in RHF [34]. In contrast, the weaker correlation observed in LHF (r = 0.8138, *p* = 0.0004) suggests that PhA may be less responsive to localized fluid retention confined primarily to the pulmonary circulation. PhA, derived from bioelectrical impedance analysis, reflects cellular integrity and hydration status and has been associated with out-comes such as nutritional status, physical performance, and mortality across various clinical populations [2,6,33]. Therefore, in the context of RHF—where systemic volume overload predominates, PhA appears to be a more reliable proxy for total body fluid distribution. In addition, the combination of PhA and plasma OSM may help as a rapid reflector for the dosage and regime of diuretics in patients with RHF which is worth further studies.

Importantly, the anatomical site of measurement significantly influences the outcomes of BIA. Human studies have demonstrated that regional BIA assessments yield variable results, as fluid distribution patterns differ across body regions [35]. In the present study, PhA was measured at the trunk, which effectively captured systemic fluid retention characteristic of RHF, particularly involving the abdomen and limbs [36]. However, in cases of LHF, where pulmonary congestion predominates [37], trunk-based measurements may fail to adequately detect thoracic fluid shifts. This limitation suggests that adapting thoracic impedance techniques, as used in human medicine, could enhance diagnostic sensitivity for pulmonary congestion in dogs. Given the anatomical and physiological variability in fluid compartments [38], future veterinary applications should explore region-specific BIA measurements to improve detection accuracy across different heart failure phenotypes.

Frequency-specific analysis also revealed meaningful physiological associations. At 5 kHz, PhA exhibited a significant negative correlation with body weight, suggesting that heavier dogs possess relatively higher extracellular fluid (ECF) volumes, leading to lower PhA values. This is consistent with animal studies, such as that by Fernandez et al. [39], which showed that while total red cell, plasma, and ECF volumes increase with body mass in rats, their normalized values (per 100 g body weight) decrease as body size increases. This inverse relationship was not observed at higher frequencies (50 kHz and 250 kHz), likely because these reflect intracellular water (ICW), which is less affected by body size. Furthermore, PhA at 50 kHz showed a significant negative correlation with age, aligning with prior findings in humans that link aging to declining cellular integrity and ICW loss [40].

Breed-specific physiological differences represent another important consideration in interpreting BIA data. Canine breeds exhibit wide variability in body composition, including disparities in muscle mass, fat distribution, and hydration status, all of which may influence impedance readings [41]. For example, sighthounds typically present lower fat mass and higher lean mass, whereas brachycephalic breeds may display the opposite. Although the InBody M20 device provides raw impedance values directly, its body composition algorithms may not be calibrated for all breeds, potentially limiting generalizability. Therefore, establishing breed-specific reference values for PhA and related parameters is essential to enhance the clinical utility and accuracy of BIA in veterinary cardiology [41].

From a practical standpoint, PhA offers several advantages over conventional imaging techniques. Compared to echocardiography or MRI, PhA assessment is faster, requires minimal training, and incurs lower operational costs. Each measurement takes less than two minutes and does not rely on consumables or laboratory infrastructure. These features make PhA particularly suitable for high-throughput clinical settings or primary care environments.

## 5. Limitations

The cross-sectional design of this study precludes assessment of temporal changes in PhA or OSM. Additionally, sample distribution across groups was uneven, and the exclusive use of trunk measurements may have limited the detection of thoracic fluid accumulation, particularly in LHF cases. LHF in dogs typically leads to pulmonary congestion and pulmonary edema due to elevated pulmonary capillary pressures, whereas pleural effusion is more commonly associated with severe right-sided or biventricular heart failure [42]. Trunk-only impedance may underestimate these region-specific fluid shifts. Finally, although strong associations were identified between PhA, OSM, body weight, and age, causality cannot be established. Future research should adopt longitudinal designs, incorporate thoracic impedance monitoring, and apply multi-frequency BIA protocols to enhance diagnostic precision and prognostic value.

A further limitation of this study is the lack of gender-based analysis. Although some human studies have reported that phase angle may vary by sex [40], we did not observe clear differences in our canine data. This might be due to variability in breed, age, and disease status, which made it difficult to isolate the effect of gender. In our dataset, the influence of sex appeared to be less prominent than other clinical factors. Nonetheless, future studies with larger and more controlled populations may help clarify this issue.

Another limitation of this study is that we were unable to confirm whether dogs had voided urine prior to BIA measurement. While fasting was controlled, bladder status was not part of our protocol. Because the measurement was not limited to a specific region and involved current flow through the trunk, we cannot rule out the possibility that retained urine may have influenced the data, especially in the lower abdominal area. Although we did not observe obvious outliers that might suggest measurement error, this remains a potential source of variation. We believe this point deserves further attention in future studies that aim to interpret impedance data more precisely.

Several prior studies have demonstrated the feasibility of applying bioelectrical impedance techniques in canine patients. Although most of these investigations focused on body composition rather than cardiovascular conditions, their findings are still valuable in validating the technical foundation of BIA in veterinary medicine. For instance, bioimpedance spectroscopy (BIS) has been shown to predict fat-free mass in dogs with reasonable accuracy when compared to dual-energy X-ray absorptiometry (DXA), with reported deviations within 3.5% of reference values [43]. This method also allows for the estimation of total, extracellular, and intracellular water volumes, which may be applicable in monitoring fluid balance [43]. Lauten et al. further provided comprehensive body composition data across various dog breeds using DXA, highlighting breed- and sex-related differences that could influence impedance measurements [44]. In addition, human pediatric studies have shown that deviations from standard positioning during DXA scanning may introduce small but measurable errors in body composition results [45]. This underscores the importance of standardizing body posture and electrode placement in veterinary BIA protocols to improve measurement consistency. Collectively, these studies provide important methodological context for interpreting BIA-derived parameters and support the cautious extension of this technology to fluid assessment in heart failure cases [46].

Another limitation of this study is the lack of longitudinal follow-up data linking PhA values to clinical outcomes such as survival, hospitalization frequency, or diuretic dose adjustment. While our findings suggest that PhA may reflect systemic fluid status in dogs with right heart failure, we could not evaluate its prognostic value or its potential role in guiding therapeutic decisions. Future studies incorporating longitudinal designs and outcome-based endpoints will be essential to validate the clinical utility of PhA and other BIA-derived parameters in veterinary cardiology.

## 6. Conclusions

This study highlights the clinical potential of phase angle (PhA) measured at 5 kHz using the InBody M20 as a reliable, non-invasive marker of extracellular fluid (ECF) status in dogs. The significant reduction in PhA observed in right heart failure (RHF), coupled with its strong correlation with plasma osmolality (OSM), supports its utility in identifying systemic fluid overload. The frequency-dependent nature of PhA further revealed that low-frequency measurements are more sensitive to body weight-related fluid shifts, while mid-frequency (50 kHz) values better reflect age-associated cellular changes. Further studies are needed on specific RHF diseases and changes in PhA and OSM with diuretics regime.

## Figures and Tables

**Table 1:** Diagnostic parameters and echocardiographic thresholds for classifying cardiac diseases in study dogs.

Disease	Diagnostic Features	Doppler Parameters	Clinical Signs
Pulmonary Stenosis (PS)	Concentric hypertrophy of the right ventricular wall. Thickened pulmonary valve with doming and narrowed orifice. Turbulent outflow in RVOT and post-stenotic dilation of the main pulmonary artery. Reduced or delayed PV opening on M-mode echocardiography [13,14,15].	Pressure gradient: Mild: <50 mmHg; Moderate: 50–80 mmHg; Severe: >80 mmHg. Velocity: Mild: 2.5–3.5 m/s; Moderate: 3.5–4.5 m/s; Severe: >4.5 m/s. Acceleration Time: AT < 60 m/s suggests severe PS [14,15,16,17].	Mild: Asymptomatic or mild exercise intolerance a left basilar systolic ejection murmur (grade II–III) is detected during routine examination. Moderate: Exercise intolerance; louder murmur with possible thrill. Severe: Syncope or right heart failure; dyspnea at rest; harsh murmur [13,15,18].
Pulmonary Regurgitation (PR)	Diastolic regurgitation flow from PA into RV seen as blue jet on color Doppler. Right ventricular and atrial dilation. Main pulmonary artery dilatation in severe PR. Incomplete PV closure during diastole on M-mode [14,16].	Regurgitation velocity: Mild: <2.5 m/s; Moderate: 2.5–3.5 m/s; Severe: >3.5 m/s. Increased RV end-diastolic dimension (RVDd). Estimated diastolic PAP: Mild: <10 mmHg; Moderate: 10–60 mmHg; Severe: ≥60 mmHg [14,16].	Mild: Often asymptomatic; diastolic murmur detected incidentally. Moderate: Exercise intolerance increased respiratory rate, possible mild abdominal distension. Right-sided heart failure (ascites, hepatomegaly, jugular distension), dyspnea even at rest [19].
Tricuspid Regurgitation (TR)	Color Doppler shows systolic regurgitant jet from RV to RA (red jet). Thickened or tethered leaflets depending on organic or functional cause. RA and RV dilation. Enhanced endocardial reflection; signs of RV pressure overload may appear [14,16].	Right atrial pressure: Mild: 3 mmHg; Moderate: 5–10 mmHg; Severe: ≥15 mmHg. Right Ventricular Pressure: Mild: 20 mmHg; Moderate: 25–30 mmHg; Severe: ≥ 30 mmHg [16,20].	Mild: Usually asymptomatic; murmur detected incidentally. Moderate: Exercise intolerance, mild tachypnea, possible abdominal distension. Severe: Signs of right-sided heart failure (ascites, hepatomegaly, anorexia), jugular vein distension, dyspnea at rest [16,20].
Mitral Regurgitation (MR)	Systolic regurgitation jet from LV to LA on color Doppler. Leaflet thickening, calcification, hypomobility, or chordal rupture. Left atrial enlargement, especially in severe cases. Increased LV end-diastolic diameter due to volume overload [13,14,15].	Left Atrial Pressure: Mild: 5–12 mmHg; Moderate: 13–18 mmHg; Severe: >18 mmHg. LA/Ao: Mild: <1.5; Moderate: 1.5–2.0; Severe: >2.0 Early diastolic velocity: Mild: ≥1.2 m/s; Moderate: 1.2 m/s; Severe: <1.5 m/s E/A: Mild: <1.5; Moderate: >1.8; Severe: >2.0 [14,15,16,17].	Mild: No clinical signs; systolic murmur detected during routine check. Moderate: Exercise intolerance, mild tachypnea, delayed recovery after activity. Severe: Dyspnea at rest, cough, pulmonary edema, cardiogenic wheezing or syncope [13,15,18].
Aortic Stenosis (AS)	2D shows thickened, calcified valve leaflets with reduced excursion. High-velocity jet seen across the aortic valve on color Doppler. Decreased and delayed opening of aortic valve on M-mode [14,16].	Left Ventricular Systolic Pressure: Mild: <180 mmHg; Moderate: 180–220 mmHg; Severe: >220 mmHg. Aortic Systolic Pressure: Mild: 100–120 mmHg; Moderate: 80–100 mmHg; Severe: <80 mmHg. Transvalvular Pressure Gradient: Mild: <60 mmHg; Moderate; 60–100 mmHg; Severe: >100 mmHg. Peak Transvalvular Velocity: Mild: <3.8 m/s; Moderate: >3.8–5.0 m/s; Severe: >5.0 m/s [14,16].	Mild: Asymptomatic; systolic murmur noted during routine examination. Moderate: Exercise intolerance, mild dyspnea, intensified systolic murmur. Severe: Syncope, cardiogenic pulmonary edema, low output, or sudden death [14,16,21].
Patent Ductus Arteriosus (PDA)	Color Doppler reveals continuous high-velocity flow from aorta to pulmonary artery. Left atrial and ventricular enlargement due to volume overload. Hyperdynamic LV contraction on M-mode. Ductal opening and turbulent flow seen near the aortic arch in short-axis view [14,16,22].	Aortic Systolic Pressure: Mild: 100–120 mmHg; Moderate: 80–100 mmHg; Severe: <80 mmHg. Pulmonary Artery Systolic Pressure: Mild: <20 mmHg; Moderate: 60–100 mmHg; Severe: >100 mmHg. Pressure Gradient Across PDA: Mild: >50 mmHg; Moderate: 20–50 mmHg [14,16].	Mild: Often asymptomatic; continuous murmur noted incidentally. Moderate: Exercise intolerance, tachypnea, cough. Severe: Pulmonary edema, dyspnea, syncope, right-sided heart failure signs [14,16,22].

**Table 2:** Distribution of Canine Subjects and Inclusion in Specific Analyses.

Group	Total Cases	Used for PhA Analysis	Used for OSM Analysis	Used for Statistical Tests
RHF	35	15	16	28
LHF	35	22	22	22
Control	40	25	14	25
Total	110	62	52	75

## Data Availability

The data presented in this study are available on request from the corresponding author.

## Abbreviations

The following abbreviations are used in this manuscript:

PhA	Phase Angle
BIA	Bioelectrical Impedance Analysis
RHF	Right Heart Failure
LHF	Left Heart Failure
OSM	Osmolality
ECF	Extracellular Fluid
ICW	Intracellular Fluid
RAAS	Renin–Angiotensin–Aldosterone System
ADH	Antidiuretic Hormone
